# Ubiquitination Links DNA Damage and Repair Signaling to Cancer Metabolism

**DOI:** 10.3390/ijms24098441

**Published:** 2023-05-08

**Authors:** Seo-Young Koo, Eun-Ji Park, Hyun-Ji Noh, Su-Mi Jo, Bo-Kyoung Ko, Hyun-Jin Shin, Chang-Woo Lee

**Affiliations:** 1 Department of Molecular Cell Biology, Samsung Medical Center, Sungkyunkwan University School of Medicine, Suwon 16419, Republic of Korea; 2 Team of Radiation Convergence Research, Korea Institute of Radiological & Medical Sciences, Seoul 01812, Republic of Korea; 3 SKKU Institute for Convergence, Sungkyunkwan University, Suwon 16419, Republic of Korea

**Keywords:** DNA damage response, DNA repair, cancer metabolism, ubiquitination, E3 ligase, therapeutics

## Abstract

Changes in the DNA damage response (DDR) and cellular metabolism are two important factors that allow cancer cells to proliferate. DDR is a set of events in which DNA damage is recognized, DNA repair factors are recruited to the site of damage, the lesion is repaired, and cellular responses associated with the damage are processed. In cancer, DDR is commonly dysregulated, and the enzymes associated with DDR are prone to changes in ubiquitination. Additionally, cellular metabolism, especially glycolysis, is upregulated in cancer cells, and enzymes in this metabolic pathway are modulated by ubiquitination. The ubiquitin–proteasome system (UPS), particularly E3 ligases, act as a bridge between cellular metabolism and DDR since they regulate the enzymes associated with the two processes. Hence, the E3 ligases with high substrate specificity are considered potential therapeutic targets for treating cancer. A number of small molecule inhibitors designed to target different components of the UPS have been developed, and several have been tested in clinical trials for human use. In this review, we discuss the role of ubiquitination on overall cellular metabolism and DDR and confirm the link between them through the E3 ligases NEDD4, APC/C^CDH1^, FBXW7, and Pellino1. In addition, we present an overview of the clinically important small molecule inhibitors and implications for their practical use.

## 1. Introduction

Ubiquitination is an important type of post-translational modification (PTM) that plays an essential role in regulating the stability or activity of substrates, subsequently mediating the function of various target proteins to maintain cellular homeostasis [1]. Ubiquitination plays a vital role in many critical biological processes, including metabolic reprogramming, DNA damage repair, cell cycle, immune responses, and cell death [1]. Consequently, dysregulation of ubiquitination can lead to the development of various diseases, including cancer [2].

Metabolic reprogramming is considered a hallmark of cancer [3] as cancer cells alter key metabolic pathways, such as glycolysis, the TCA cycle, and fatty acid and amino acid metabolism, in order to promote their own growth and survival in their specific microenvironment. Signaling pathways, transcription factors, and metabolic enzymes associated with the adjustment of cancer metabolism are regulated by PTMs, particularly ubiquitination. For instance, various proteins related to AKT-PI3K-mTOR signaling are ubiquitinated by multiple E3 ligases, which regulate glucose and lipid metabolism in cancer [1,4]. Another example can be seen with transcription factor HIF-1α, promoting the expression of various glycolytic enzymes, which is stabilized by the loss of E3 ligase VHL in cancer cells and consequently contributes to aerobic glycolysis. As well, metabolic enzymes such as Hexokinase2 (HK2) either enhance or disrupt glycolysis and tumor growth depending on ubiquitination by E3 ligase, including HUWE1 and TRAF6 [3]. Recent studies have revealed that aberrant expression and activity of E3 ligases are involved in the molecular etiology and pathogenesis of cancer development and progression [1]. Given that multiple ubiquitination targets mediate cancer metabolism, understanding the role of E3 ligases is important to gain insight into the mechanisms underlying metabolic reprogramming in cancer.

The DNA damage response (DDR) is a crucial mechanism for maintaining genomic integrity as well as cell fitness. DNA double-strand break (DSB) repair pathways are activated and regulated by E3 ligases, such as RNF8 and RNF168. Both of these ubiquitinate histone H2A and H2AX, leading to the recruitment of DNA repair factors and activation of DSB signaling [5]. In addition, ubiquitination in DDR has an important role in understanding DNA repair mechanisms depending on the type of DNA damage and cellular condition [6]. For instance, ubiquitination plays a role in determining between the two major repair pathways for double-strand break (DSB) DNA damage: non-homologous end joining (NHEJ) and homologous recombination (HR) [7]. DSB end resection, an initial repair process of HR, is inhibited by Ku70–Ku80 heterodimer (Ku), and the timing of ubiquitination and subsequent removal of Ku from the DSB is a critical determinant for choosing the NHEJ or HR pathway [6]. Ubiquitination is also involved in repairing DNA lesions as well as choosing the repair pathway. By regulating the activity and stability of repair proteins, a sophisticated DNA damage repair process can be orchestrated, allowing precise control over timing and localization. Understanding the mechanism of E3 ligases-mediated DDR is crucial for elucidating DNA damage and repair.

Links between DNA damage and cancer metabolism are becoming progressively clearer [8]. However, the mechanisms that connect both DDR and metabolic reprogramming are still unknown. We believe that both the DNA damage response and cancer metabolism are regulated by ubiquitination since a target substrate that is ubiquitinated by E3 ligase affects these two pathways. Given that multiple ubiquitinations simultaneously regulate these processes, this review suggests that ubiquitination, particularly via E3 ligase, is a critical regulator linking DNA damage and cellular metabolism in cancer cells.

Recently, anti-cancer treatments targeting E3 ligase have been actively developed, and the therapeutic effects of various agents have been investigated by preclinical studies as well as clinical trials [1,9]. Several studies have revealed that combining of E3 ligase inhibitor with radiation therapy inhibits tumor growth with high efficiency [10]. Also, some E3 ligase inhibitors induce apoptosis in tumors by inhibiting the interaction of E3 ligase with proteins related to cancer metabolism [11]. Understanding the role of E3 ligases as a linker between DNA damage repair and cancer metabolism offers new therapeutic strategies. In addition, since E3 ligase generates specificity in the ubiquitination system, more elaborate regulation of DDR and metabolic reprogramming is possible, and from this, expectations of more sophisticated cancer therapy will follow. This review introduces the concept that E3 ligase plays a role in connecting DNA damage to metabolic reprogramming and suggests applying it to clinical trials in cancer therapy.

## 2. The Effects of Ubiquitination on Cancer Metabolism

Cellular metabolism is a set of biochemical reactions that sustain life in an organism. Cellular metabolism is altered in cancer cells to meet increased bioenergetic and biosynthetic demands as well as to alleviate oxidative stress for their survival and proliferation [12]. The classic example of altered metabolism in cancer cells is aerobic glycolysis or the Warburg effect, which manifests as an increase in glucose uptake and lactate production even in the presence of oxygen [12,13]. It has also been demonstrated that the TCA cycle is critical to certain cancer cells for energy production and macromolecule biosynthesis despite the long-standing belief that cancer cells primarily use aerobic glycolysis and bypass the TCA cycle [14]. Fatty acid and amino acid metabolism are upregulated in cancer as well. There are a number of substrate proteins involved in cellular metabolism, and these are precisely regulated by ubiquitination [2]. Therefore, dysregulated ubiquitination of proteins can lead to the onset and progression of disease [2]. Indeed, dysfunctional ubiquitination has been observed in various types of cancer [3].

Ubiquitination takes place in three enzymatic steps [1]. First, the ubiquitin-activating enzyme (E1) activates ubiquitin in an ATP-dependent manner. E1 enzyme then transfers the activated ubiquitin to the ubiquitin-conjugating enzyme (E2). Finally, ubiquitin ligase (E3) transfers ubiquitin from E2 to substrate [15]. There are two main subtypes of E3 ubiquitin ligases in humans, as defined by the presence of either homologous to the E6AP carboxyl terminus (HECT) domain or really interesting new gene (RING) finger domain on the basis of structural similitude. Monoubiquitination is the attachment of a single ubiquitin to a specific lysine residue on proteins, while polyubiquitination, as the name suggests, is the attachment of a chain of ubiquitin molecules to a lysine residue [3]. There are multiple types of ubiquitin chains depending on the lysine residue in the substrate (K6, K11, K27, K29, K33, K48, and K63) [15]. Among them, K48- and K63-linked polyubiquitination have been the most widely studied, and the former results in 26S proteasome-mediated degradation, while the latter results in protein stabilization and activation [2,15]. Since ubiquitination plays a significant role in the regulation of metabolic enzymes, dysregulated ubiquitination can promote the metabolic reprogramming of cancer and tumor growth [3].

Glycolysis is a catabolic process that splits glucose into two molecules of pyruvate to release energy under oxygen-free conditions [13]. It is less efficient than oxidative phosphorylation (OXPHOS) in terms of ATP production, but cancer cells utilize glycolysis to supply more glycolytic intermediates for biosynthetic pathways [16]. Additionally, the metabolic enzymes and transcription factors involved in glycolysis are prone to ubiquitination. For example, HK2, which catalyzes the phosphorylation of glucose to glucose 6-phosphate (G6P) in the first rate-limiting reaction of glycolysis, is ubiquitinated by TRAF6 and HUWE1 [17,18]. The E3 ligase HUWE1 mediates the K63-linked ubiquitination of HK2, which leads to its re-localization and activation [18]. The activated HK2 enhances aerobic glycolysis and tumorigenesis as a key regulator of glucose metabolism, promoting a glycolytic switch from OXPHOS to aerobic glycolysis [19]. Similarly, phosphofructokinase (PFK), which serves to phosphorylate fructose-6-phosphate (F6P) in the second rate-limiting step of glycolysis, is ubiquitinated by TRIM21 and A20 [20,21]. The E3 ubiquitin ligase TRIM21 mediates ubiquitination and degradation of PFK1 but is downregulated in some cancers, including hepatocellular carcinoma and non-small cell lung cancer (NSCLC) [20,22]. The overexpressed PFK1 augments glycolysis to promote tumor growth [23]. Pyruvate kinase M2 (PKM2) is required for the last step of glycolysis and is ubiquitinated by Parkin, CHIP, and TRIM58 [24,25,26]. TRIM58, which ubiquitinates and destabilizes PKM2 [26], has been reported to be downregulated in several types of cancers, including liver, lung, colorectal cancers, and osteosarcoma [26,27,28,29], resulting in PKM2 accumulation and increased aerobic glycolysis [26]. Another glycolysis enzyme, phosphoglycerate kinase 1 (PGK1), which catalyzes the conversion of 1,3-bisphosphoglycerate (1,3BPG) to 3-phosphoglycerate (3PG), also undergoes ubiquitination and subsequent degradation by STUB1 [30]. In gallbladder cancer cells, downregulated STUB1 leads to the overexpression of PGK1, followed by cancer proliferation and metastasis [31]. PGK1 has multi-faceted roles in addition to cell metabolism regulation and is correlated with chemotherapy resistance and poor prognosis in most cancers [32]. GLUT1 is a glucose transporter that facilitates glucose uptake across the plasma membrane [33]. It is under the control of the master regulator AKT, which is ubiquitinated by TRAF6 and Skp2 [34]. The E3 ligase Skp2 ubiquitinates and activates AKT, which promotes the expression of downstream proteins, including glucose transporters and glycolytic enzymes, thus supporting glycolysis and tumor growth [4,35].

The TCA cycle is a metabolic pathway that generates cellular energy and precursors for other biosynthetic pathways, such as fatty acid and amino acid synthesis, as well as gluconeogenesis [36]. Cancer cells prefer glycolysis over mitochondrial respiration, but they still have functional mitochondria for OXPHOS [36]. Hence, the dysregulation of metabolic enzymes in the TCA cycle through ubiquitination can affect tumorigenesis. Citrate synthase (CS), which plays a role in catalyzing the condensation of acetyl CoA and oxaloacetate (OA) to generate citrate, is regulated through ubiquitination by UBR5 and SCF^Ucc1^ [37,38]. UBR5-facilitated ubiquitination of CS leads to its degradation at the posttranslational level [38]. In hypoxic breast cancer cells, the ubiquitination and degradation of CS are diminished, resulting in elevated CS activity [38]. The activated CS increases the production and export of citrate for lipid biosynthesis, which promotes cell migration, invasion, and metastasis [38]. In fact, the role of CS in tumor cell growth is complicated and cancer-type specific, as seen by the fact that it is upregulated in pancreatic, renal, and ovarian cancers but is downregulated in some cervical cancers [39]. This is most likely explained by different metabolic requirements since pancreatic cancers depend primarily on lipid synthesis to proliferate, while cervical cancers rely more on glycolysis [40]. Furthermore, isocitrate dehydrogenases 1 and 2 (IDH1/2), which catalyze the conversion of isocitrate to α-ketoglutarate (α-KG), are both abnormally regulated in cancer [41]. IDH1 is indirectly regulated by FBXW7, which mediates the ubiquitination and degradation of SREBP1 [42]. In human gliomas, FBXW7 is downregulated, which inhibits the degradation of SREBP1, which in turn, increases the expression of IDH1 [42]. IDH1 decreases the cellular buffering ability against radiation-induced oxidative stress, thus enhancing the radiation sensitivity of gliomas [42]. In addition, IDH2 is ubiquitinated and degraded by APC/C^Cdh1^, contributing to an increase in reactive oxygen species (ROS) levels during mitosis [43]. IDH2 generates NADPH, which is a cofactor used by ROS-scavenging enzymes to block ROS production in cells [43]. α-Ketoglutarate dehydrogenase (α-KGDH) catalyzes the conversion of α-KG to succinate and is ubiquitinated and degraded by SIAH2 under hypoxic conditions [44]. The inhibition of α-KGDH leads to an imbalance in the α-KG/citrate ratio and TCA cycle functioning in the reverse direction [45]. This promotes de novo fatty acid synthesis but ultimately impedes tumor growth as cancer cells become dependent on citrate or exogenous lipids to proliferate [45]. HIF-1α is a transcription factor that regulates the expression of many glycolytic enzymes and thus is important in tumorigenesis [46]. The E3 ligase pVHL mediates the ubiquitination and degradation of HIF-1α [47]. However, reduced succinate dehydrogenase (SDH) activity leads to succinate accumulation in cells which inhibits hydroxylation of HIF-1α, and thus leads to thedissociation of pVHL from HIF-1α [48]. Stabilized HIF-1α activates the glycolytic enzymes and cancer progression [46,48].

Changes in lipid metabolism are crucial for cancer progression because fatty acids are required for producing energy, signaling molecules, and cell membranes [49]. Lipid metabolism is largely divided into fatty acid oxidation (FAO), fatty acid synthesis (FAS), and de novo lipogenesis (DNL) [50]. FAO is the process by which fatty acids are broken down to produce acetyl CoA, which can either be used to feed the TCA cycle or for the synthesis of fatty acids. FAS is the opposite of the FAO pathway in that fatty acids are synthesized from acetyl CoA [50]. Lastly, the DNL is an endogenous pathway that converts excess carbohydrates into fatty acids [51]. There are numerous enzymes involved in lipid metabolism, many of which are affected by ubiquitination. For example, the CPT2 protein serves to transfer fatty acids across the inner mitochondrial membrane for β-oxidation and undergoes K48-linked ubiquitination and degradation by HRD1 [52]. In glutamine-deficient triple-negative breast cancer (TNBC), HRD1 expression is significantly downregulated, resulting in the increase of CPT2 [52]. As TNBC cells depend on glutamine and use fatty acids as an alternative energy source under conditions of glutamine deprivation [52], HRD1 deregulation in TNBC can contribute to the survival of cancer by supporting lipid metabolism. Additionally, upregulated FAO supports cancer cell growth, stemness, drug resistance, and metastasis [53]. ATP citrate lyase (ACLY) and fatty acid synthase (FASN) play significant roles in the FAS pathway, as citric acid is converted to acetyl CoA via ACLY and subsequently to fatty acids via FASN [54]. The deubiquitinating enzyme (DUB) USP13 deubiquitinates and stabilizes ACLY and is overexpressed in highly invasive ovarian cancer (OVCA) [55]. Activated ACLY is correlated with malignant development and poor prognosis of OVCA [55]. In addition, USP30 deubiquitinates and stabilizes ACLY and FASN and is overexpressed in the high-fat diet (HFD)-induced hepatocellular carcinoma (HCC) [56]. The upregulation of ACLY and FASN promotes tumorigenesis by enhancing lipid synthesis [56,57]. SREBP1C is a master transcription factor that regulates the expression of lipogenesis-associated genes [58]. USP7 deubiquitinates and stabilizes ZNF638, and USP7/ZNF638 axis increases the cleavage of SREBP1C through AKT/mTORC1/S6K signaling [59]. Hence, USP7/ZNF638/SREBP1C complex upregulates the expression of acetyl CoA carboxylase (ACACA), FASN, and stearoyl-CoA desaturase (SCD) to promote DNL and tumor initiation [59].

Amino acid metabolism is interconnected with other metabolic pathways and is involved in the biosynthesis of lipid and nucleic acid as well as providing building blocks for protein synthesis [60]. In particular, glutamine is one of the most important nutrients in cancer cells because it is necessary for TCA cycle supplementation, nonessential amino acid (NEAA) synthesis, nucleic acid synthesis, and the generation of glutathione (GSH) and NADPH that counteract oxidative stress [61]. Glutamine uptake occurs via the neutral amino acid transporter ASCT2, which undergoes ubiquitination and degradation by NEDD4L [62]. Therefore, NEDD4L-deficient cancer cells exhibit increased levels of ASCT2 and glutamine transport, enhancing mitochondrial respiration and tumorigenesis [62]. The transported glutamine undergoes glutaminolysis, which begins with the deamination of glutamine via glutaminase (GLS) into glutamate [63]. The desuccinylation of GLS at Lys158 and Lys164 sites by SIRT5 enables its K48-linked ubiquitination and degradation [63]. The BAG3 protein prevents interaction between GLS and SIRT5, resulting in the succinylation of GLS, thereby inhibiting ubiquitination and proteolysis [63]. In turn, glutaminolysis is promoted, and glutamate is further converted to α-KG to fuel the TCA cycle. Additionally, D-3-phosphoglycerate dehydrogenase (PHGDH) catalyzes the synthesis of serine from the intermediate product of glycolysis, 3PG, and the E3 ligase PARKIN mediates its ubiquitination and subsequent degradation. Therefore, the downregulated PARKIN in cancer cells stabilizes PHGDH and enhances serine synthesis and tumor proliferation [64]. Serine serves as a source of the one-carbon unit for nucleotide synthesis and DNA methylation, and it is further converted to glycine by serine hydroxymethyltransferase 1 (SHMT1) [65,66]. UBC13 facilitates the K63-linked ubiquitination of SHMT1, which promotes its nuclear export and stabilization [67]. The activated SHMT1 increases glycine synthesis and tumorigenesis, as glycine is a key metabolite [68]. Moreover, glutamine synthetase (GS) catalyzes the conversion of glutamate and ammonia into glutamine, and Cullin-RING ubiquitin ligase 4 (CRL4) facilitates the ubiquitination and proteasomal degradation of GS [69]. However, USP15 antagonizes the CRL4-mediated ubiquitination of GS and inhibits its degradation [70]. USP15 is overexpressed in immunomodulatory drug (IMiD)-resistant cells, and targeting USP15 sensitizes these cells to IMiD, indicating that ubiquitination is important in cancer [70].

Metabolic reprogramming is considered one of the key features of cancer cells. Metabolic enzymes, transcription factors (TFs), and signaling pathways are all involved in the modulation of cancer metabolism and are regulated by ubiquitination and deubiquitination [3]. Therefore, aberrant regulation of ubiquitination and deubiquitination can lead to the onset and progression of diseases such as cancer. Since ubiquitination regulates proteins with key functions in cancer, many small molecule inhibitors targeting the ubiquitin–proteasome system (UPS), including the proteasome, E3 ligases, E1, E2, and DUBs, have been developed, and some are undergoing clinical trials [1]. However, there are currently limitations that inhibit their widespread applications in humans. As our understanding of the full role of targeted proteins involved in cancer metabolism and their effect on other cellular activities are not entirely clear, elucidating their specific roles and interactions with other cellular processes will be necessary for the search to find effective therapeutic targets.

## 3. The Effects of Ubiquitination on DNA Damage and DNA Repair

Immediate and accurate DDR is crucial to maintaining the integrity of the genome since DNA constantly suffers exogenous or endogenous damage [7]. Upon DNA damage, DDR induces cell cycle arrest at a certain stage and permits the repair of damaged DNA [71]. PTMs (e.g., phosphorylation, ubiquitination, and SUMOylation) of chromatin and chromatin-associated proteins are triggered by the presence of damaged DNA [72]. Of all the PTMs, ubiquitination is an especially important modification in the DNA repair pathway, as ubiquitination is highly prevalent at DNA break sites and acts to orchestrate the appropriate DNA repair pathway by providing recruitment signals for DNA repair proteins or by stimulating proteasomal degradation to regulate their expression levels [72]. Initially, the DNA repair pathway starts with the recruitment of DNA repair factors at single-strand break (SSB) and DSB sites. This process is regulated by ubiquitination through the E3 ubiquitin ligase RNF8-RNF168 pathway, which is the central mediator of chromatin-associated ubiquitination. RNF8 and RNF168 ubiquitinate histone and promote the recruitment of downstream factors, especially p53-binding protein 1 (53BP1) and breast cancer type 1 susceptibility protein (BRCA1), which are important for DNA repair pathway choice. DSBs are mainly repaired by HR or NHEJ, and the choice of which pathway is determined by the expression of distinct protein complexes [71]. Ubiquitination and deubiquitination regulate the expression of protein complexes, allowing them to function differently depending on the cell cycle [73].

**Figure 1:**
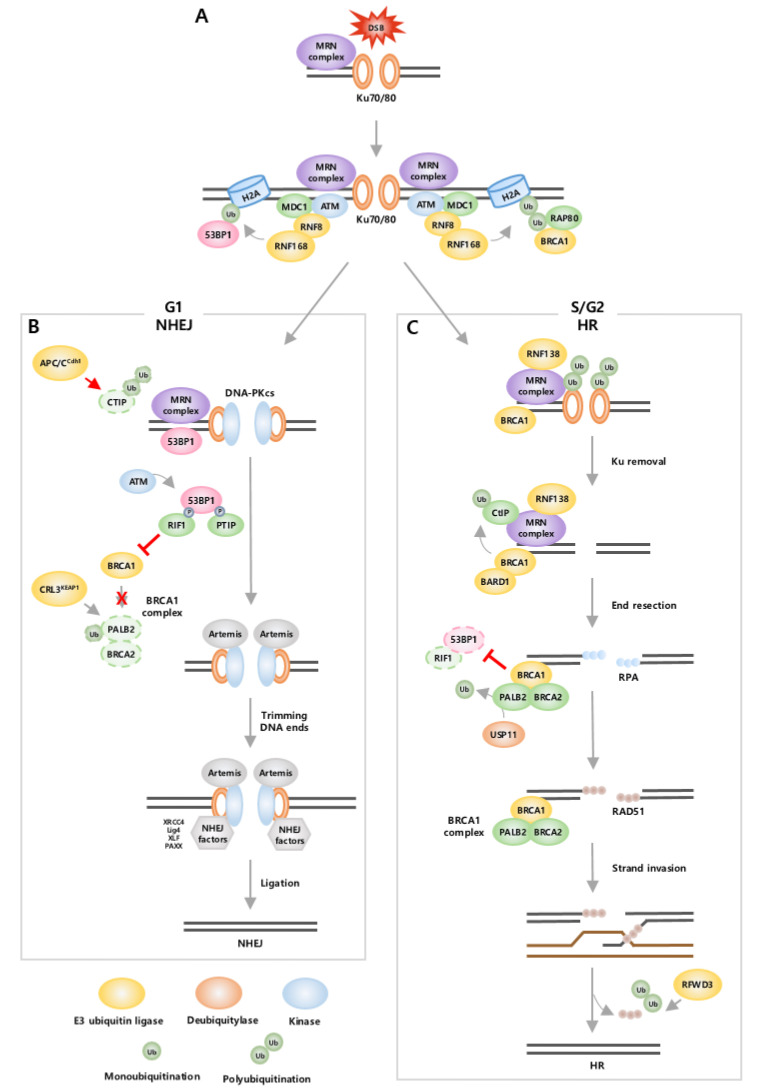
Ubiquitination regulates DSB repair pathway. (**A**) DSBs can be repaired by NHEJ and HR. Both pathways initiate with recruitment of MRN complex and E3 ubiquitin ligases RNF8 and RNF168. DNA repair pathway choice is determined by recruitment of 53BP1 or BRCA1 in H2A. 53BP1 leads NHEJ in G1 phase, whereas BRCA1 leads HR in S/G2 phase. (**B**) In NHEJ, Ku recruits DNA-PKcs. 53BP1 that phosphorylated by ATM binds with PTIP and RIF1. 53BP1 and PTIP recruit Artemis, which trims the DNA ends. Additional NHEJ factors assemble and ligate DNA breaks. During NHEJ, HR is suppressed through ubiquitin-mediated degradation of CtIP by APC/C^Cdh1^ and inhibition of BRCA1 by 53BP1-RIF1 and CRL3^KEAP1^. (**C**) In HR, RNF138 ubiquitinates Ku for degradation. BRCA1, MRN, and CtIP form a complex, and ubiquitinated CtIP takes part in end resection. Then, ssDNA tail is bound by RPA. When PALB2 is deubiquitinated by USP11, BRCA1-PALB2-BRCA2 complex mediate replacement of RPA into RAD51. RAD51-coated filaments invade the homologous strand. Finally, DNA synthesis is completed with ubiquitin-mediated degradation of RAD51 by RFWD3. During HR, NHEJ is suppressed through inhibition of 53BP1 by BRCA1.

The first step in the DNA repair pathway is the recruitment of repair factors at DNA break sites [6], which are initially recognized by the MRN (MRE11-RAD50-NBS1) complex. NBS1, a component of the MRN complex, is essential for ataxia telangiectasia mutated (ATM) kinase recruitment to DSB sites [74]. K63-linked ubiquitination of NBS1 by E3 ligase Pellino1 (Peli1) and Skp2 promotes stable maintenance of the MRN complex and facilitates further activation of ATM [75,76,77,78]. Subsequently, activated ATM phosphorylates the histone H2A variant H2AX at S139 (known as γH2AX, the phosphorylated form of H2AX), creating a binding site for the scaffold protein, Mediator of DNA Damage Checkpoint 1 (MDC1) [6]. After MDC1 binds to γH2AX [79], MDC1 recruits RNF8 and E2 ubiquitin-conjugating enzyme, Ubc13. RNF8, together with Ubc13, promotes K63-linked polyubiquitination of H1-type linker histones and then recruits another E3 ubiquitin ligase RNF168 to DSB sites [80]. There is also an alternative pathway to recruit RNF8 and RNF168. MDC1 recruits Lethal (3) malignant brain tumor-like protein 2 (L3MBTL2), which is subsequently ubiquitinated by RNF8, resulting in the binding of RNF168 to DSB sites [81,82]. RNF8 and RNF168 then ubiquitinate H2A, triggering the recruitment of many important repair proteins, including 53BP1 and BRCA1 [81]. When RNF168 mediates monoubiquitination of H2A at K13/15, 53BP1 binds directly and selectively to H2AK15ub and is recruited to DSB sites [83]. When monoubiquitination is extended with K63-linked ubiquitin chains by RNF8, RAP80 interacts with the K63-linked ubiquitin chain and leads to the recruitment of a BRCA1-A complex (including BRCA1, RAP80, BRCC36, BRCC45, MERIT40, and Abraxas) (Figure 1A). In the normal state, the activity of the RNF8-RNF168 pathway is tightly regulated to prevent their hyperaccumulation, as this leads to excessive spreading of histone ubiquitination, increased recruitment of 53BP1 and BRCA1, and DNA repair alteration. Hence, the expression level of RNF168 is limited through degradation by E3 ubiquitin ligases TRIP12 and UBR5 [84]. In addition, DUBs are also involved in negatively regulating the RNF8/RNF168 pathway to suppress excessive histone ubiquitination. USP44 directly removes RNF168-mediated H2A ubiquitination at K13/15. OTU deubiquitinase, ubiquitin aldehyde binding 1 (OTUB1) inhibits the Ubc13 activity that catalyzes K63-linked polyubiquitination of H2A together with RNF8 and RNF168 [82,85,86].

The recruitment of 53BP1 or BRCA1 plays an important role in determining the choice of which DSB repair pathway to use, NHEJ or HR. NHEJ is an error-prone pathway since it directly ligates two broken DNA ends, and it is activated throughout the cell cycle, primarily in the G1 phase. During NHEJ, 53BP1 is stimulated, and BRCA1 recruitment is inhibited from suppressing HR. In contrast, HR is an accurate pathway as it goes through end resection for the degradation of damaged DNA ends and uses the homologous DNA strand as a template for repair. Hence, HR is restrained in the G1 phase and activated only in the S/G2 phase when the sister chromatid is available as a template [87]. During HR, BRCA1 is stimulated and inhibits the recruitment of 53BP1 to suppress NHEJ [88]. Consequently, 53BP1 and BRCA1 act antagonistically and activate distinct complex signaling cascades through regulation by ubiquitination [88].

In the G1 phase, NHEJ starts with recognition of the DNA ends by Ku70-80 heterodimer (Ku), which leads to the recruitment of DNA-dependent protein kinase catalytic subunit (DNA-PKcs) [89,90]. DNA-PKcs serve as a platform to recruit DNA end-processing nuclease Artemis, 53BP1, and other NHEJ factors [90]. 53BP1 phosphorylated by ATM binds to the downstream mediators, PAX transactivation domain-interacting protein (PTIP) and RAP1 Interacting Factor Homolog (RIF1) [91]. PTIP, together with 53BP1, recruits Artemis to trim the DNA ends, and subsequently, the DNA break is connected [6]. Mechanisms exist to inhibit HR-related factors in the G1 phase to restrain HR [92]. 53BP1 and RIF1 cooperate to inhibit recruitment of BRCA1 in DSB sites, thereby restricting BRCA1 to function only in S/G2 phase. E3 ligase complex CRL3^KEAP1^ ubiquitinates partner and localizer of BRCA2 (PALB2), a component of BRCA1 complex (including BRCA1, PALB2, BRCA2, and RAD51 [93]), to limit its ability to promote HR. Specifically, this process inhibits the recruitment of BRCA2 at DSB sites by preventing the interaction of BRCA1 with PALB2-BRCA2 [92,94]. In addition, E3 ubiquitin ligase APC/C^Cdh1^ and SIAH-1 mediate ubiquitination and degradation of CtBP-interacting protein (CtIP), thus inhibiting mediating DNA end resection [95] (Figure 1B).

In the S/G2 phase, BRCA1 is the main factor that mediates HR, and this occurs through the formation of several functionally distinct BRCA1 complexes [83]. HR is processed by removal of Ku, end resection, strand invasion, and DNA synthesis [89,96]. In contrast to NHEJ, Ku needs to be removed from the DNA end to generate an ssDNA tail. This process is mediated by E3 ubiquitin ligase RNF138, which ubiquitinates the Ku80 subunit for proteasomal degradation, allowing end resection factors to recruit into DSB sites [6]. Next, DNA end resection is initiated by CtIP within the BRCA1-C complex (including BRCA1, CtIP, and MRN). CtIP is ubiquitinated by E3 ubiquitin ligase BRCA1/BRCA1-associated RING domain 1 (BARD1) complex [97], in which BRCA1 imparts E3 ubiquitin ligase activity through heterodimerization with BARD1 [98,99]. Ubiquitinated CtIP is recruited to DSB sites [97] and interacts with the MRN complex to generate a short ssDNA tail, which is rapidly bound by replication protein A (RPA) [100,101]. Then, RPA is replaced with RAD51 to promote strand invasion on the homologous template with the help of the BRCA1 complex (including BRCA1, PALB2, BRCA2, and RAD51). To achieve this process, PALB2 is first deubiquitinated by USP11 to form the BRCA1 complex [93]. Then, BRCA2 within the BRCA1 complex recruits and promotes RAD51 loading on ssDNA at sites of DSBs [102]. However, to synthesize the DNA strand and terminate HR, RAD51 is removed through polyubiquitination by E3 ligase RFWD3 for proteasomal degradation [89,93,96]. Furthermore, BRCA1 also functions to antagonize NHEJ by inhibiting two essential NHEJ regulators, 53BR1 and RIF1, in S and G2 phases. For example, BRCA1/BARD1 complex ubiquitinates H2A at K127/K129, leading to removal of 53BP1 from DSB sites [83,103]. BRCA1 recruits E3 ligase UHRF1, which then mediates K63-linked polyubiquitination of RIF1, promoting RIF1 dissociation from DSB sites [82,88] (Figure 1C).

Many studies have reported that dysregulation of DNA repair pathways and ubiquitin-dependent DSB signaling is highly associated with the initiation and progression of cancer [96]. Therefore, cancers that develop through these pathways exhibit vulnerability to specific DNA-damaging drugs or radiation therapy. However, in many cancers, DDR pathways are hyperactivated, and perturbed DNA repair pathways are promoted to resist these therapies, allowing tumor cells to survive [8,104]. Dysregulated DNA repair is significantly implicated in ubiquitin pathways since DNA repair factors regulated by ubiquitin pathways are often mutated and either highly over- or under-expressed in tumor cells. In addition, there is growing evidence that E3 ubiquitin ligases link hyperactivated DDR pathways with cancer metabolism by mediating both processes [8]. Therefore, the DNA repair mechanism and its ubiquitin-mediated pathways offer promising targets for novel cancer therapies. Several DNA repair inhibitors, such as PARP inhibitors and ATM inhibitors, have been developed, and clinical trials are ongoing [105]. Moreover, there have been attempts to develop DNA repair inhibitors to use in combination therapy since impaired DNA repair increases the efficacy of DNA-damaging anti-cancer drugs [104]. Recently, much work has been directed toward investigating the therapeutic potential of regulating E1, E2, and E3 enzymes, DUBs, and UPS, and there has been significant progress in developing inhibitors targeting ubiquitin cascades [106]. As targeting of DNA repair pathways has shown strong potential for cancer treatment [105], a better understanding of how ubiquitination is associated with DDR and DNA repair pathway and its underlying mechanisms in cancer will offer new therapeutic opportunities for cancer treatment [104].

## 4. E3 Ligase Involved in Cancer Metabolism and DNA Damage

Cellular metabolism can either negatively or positively affect genomic integrity by leading to DNA damage or by facilitating DDR pathways [107]. ROS is elevated in cancer as a consequence of metabolic reprogramming, which increases oxidative DNA damage. The accumulation of oxidative DNA damage enhances the load on DNA repair [108]. In addition, cancer cells exhibit de novo nucleotide synthesis, which has an impact on the pool of nucleotides used for DNA replication and repair. Essential precursors of the bases that constitute nucleotides come from the intermediate of the pentose–phosphate pathway (PPP) mechanism, which supports cancer cell survival and growth and generates NADPH required for biosynthesis for nucleotides [108,109]. Interestingly, while metabolism affects DNA lesions and repair, DNA damage triggers metabolic rewiring [107]. Several studies have shown that DDR factors, such as ATM and DNA-PK, not only recognize DNA damage and induce DNA repair signaling but are also involved in cellular metabolism rewiring after DNA damage [8]. For instance, ATM activates AKT and subsequently triggers glucose recruitment via GLUT4-mediated transport. Similar to ATM, DNA-PK regulates AMP-activated protein kinase (AMPK), followed by increased glycolysis [8].

E3 ligases confer specificity on the overall process of ubiquitination through distinct ubiquitination mechanisms and recognition of certain substrates [110]. Based on catalytic mechanisms, E3 ligases can be divided into three subgroups: HECT-type, RING-type, and RING-between-RING (RBR)-type [110]. HECT ligases catalyze substrate ubiquitination in a two-step reaction, whereas RING ligases directly transfer ubiquitin from E2 to a substrate. A RING/HECT hybrid mechanism occurs by RBR ligases, performing a multi-step reaction with RING1 and RING2 [111]. Further specificity comes from specific molecular recognition of ubiquitin ligase. As the last component of the ubiquitination cascade, E3 ligases have the substrate-targeting subunit, which recognizes and binds to the degron, which is a short linear motif in the target protein [112]. Since specificity in ubiquitination is generated by E3 ligases, regulation of E3 ligases enables the delicate modification of biological processes.

Multiple studies have shown that various E3 ligases are engaged in DDR and metabolic reprogramming (Table 1 and Table 2). Since several ubiquitin ligases contribute to both processes, E3 ligases are expected to function as linkers between DNA damage repair and cancer metabolism. There are two possibilities in terms of how E3 ligase contributes to these two pathways. First, target substrates by regulating E3 ligase affect metabolic progress and DNA repair in cancer cells. For example, ACLY is ubiquitinated and subsequently degraded by E3 ligase NEDD4 in lung cancer cells and is a key metabolic enzyme that produces the acetyl-CoA required for fatty acid metabolism in cancer [113,114]. In NSCLC, the stability and activation of ACLY are enhanced by the dissociation between E3 ligase NEDD4 and ACLY, which promotes a high level of acetyl-CoA and fatty metabolism, subsequently inducing tumor proliferation [115]. ACLY also participates in DDR by promoting histone acetylation, which is important for the proper repair of DNA DSBs in response to DNA damage [116]. Given that ACLY produces acetyl CoA to induce fatty acid metabolism in cancer cells by dissociation from NEDD4 and also facilitates histone acetylation in response to DNA damage, while NEDD4 affects various pathways involved in DDR, the regulation of NEDD4 is likely to play a role as an important link between DNA repair and cancer metabolism. Another possibility is that E3 ligases induce the modification of both DNA repair and cancer metabolism. For instance, E3 ligase BRCA1 is well known to play an essential role in DNA damage repair, especially HR, by ubiquitination of histone protein H2A [117]. Histone ubiquitination is recognized by DDR proteins, triggers the recruitment of repair proteins to DNA lesion sites, and eventually facilitates the DNA repair pathway [83]. BRCA1 also controls cancer metabolism by ubiquitination of AKT, eventually suppressing oncogenesis. BRCA1 directly interacts with AKT and downregulates its activity through ubiquitination-mediated degradation [3]. Following this, aberrant activation of PI3K/AKT alters metabolic reprogramming related to glycolysis in various pathways. In addition to BRCA1, highly expressed E3 ligase in specific cancer cells is likely to cross-connect DDR to cancer metabolism. Recent studies have shown that in esophageal squamous cell carcinoma (ESCC), the expression of RNF168 was increased, which enhances tumor growth [118]. Moreover, mechanistic studies show that RNF168 is positively correlated with WNT3A, β-catenin, and glycogen synthase kinase 3β (GSK-3β) expression, all of which are involved in the Wnt/β-catenin signaling pathway. Since several studies have observed that the Wnt/β-catenin signaling pathway upregulates the expression of glycolytic enzymes, abundant RNF168 triggers glycolysis in tumor cells [119]. Overexpression of RNF168 in tumor cells also induces abnormal DNA repair, imbalancing this repair pathway and resulting in cancer [118]. Specifically, in the S phase, high levels of RNF168 fuel aberrant 53BP1 recruitment, not BRCA1, subsequently promotes mutagenic NHEJ, leading to genomic instability [120]. If the expression of RNF168 is downregulated, the DNA repair pathway is balanced and signaling related to glycolysis is inhibited, leading to the suppression of tumorigenesis. Similar to these examples, it is possible that other E3 ligases regulate both. Understanding the function of ubiquitin ligase in DDR and metabolic reprogramming will provide insight into the role of E3 ligase as a linker between the two processes. More evidence that E3 ligases contribute to both, and direct experiments to determine which candidates from among the family of E3 ligases perform these functions, are needed.

As noted, while growing evidence has shown that DNA damage and repair interplay with metabolic reprogramming, studies on the upstream regulation of these linkages have yet to be appreciably explored [108]. If it is accepted that E3 ligase mediates this relationship, then it offers a tempting therapeutic target for regulating both DNA repair and cancer metabolism. Therefore, intensifying efforts aimed at understanding E3 ligases, and the crosstalk between DNA damage and repair with cancer metabolism, will undoubtedly cast new light on the therapeutic options. For more details, the following sections introduce representative E3 ubiquitin ligases that contribute to both DNA damage response and cancer metabolism. We selected representative proteins, NEDD4, APC/CCDH1, and FBXW7, which have been extensively studied in cancer metabolism and are also involved in the DDR pathway. Additionally, we introduce E3 ubiquitin ligase Pellino1, which has been well-studied for DDR and cancer metabolism, respectively, as a novel linker of these two pathways.

### 4.1. NEDD4

**Figure 2:**
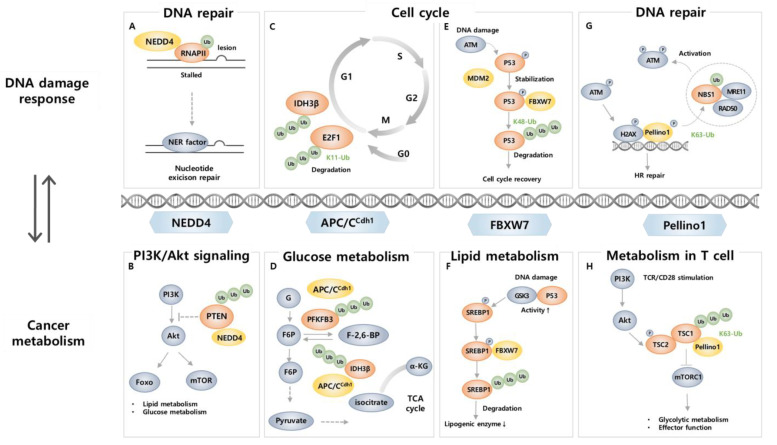
E3 ubiquitin ligases link DDR-related proteins and cancer metabolism-related proteins. (**A**) RNAPII functionally stalled at the DNA lesion is ubiquitinated by NEDD4 for proteasomal degradation, allowing DNA repair and restarting transcription. (**B**) PTEN acts as an antagonist to PI3K; however, NEDD4 ubiquitinates PTEN to downregulate its activity, allowing PI3K/AKT signaling. (**C**) E2F1 accumulation during G1/S transition is controlled by APC/C^cdh1^ mediated ubiquitination. (**D**) IDH3β and PFKFB3, related to glucose metabolism, undergo ubiquitination-mediated degradation by APC/C^cdh1^. (**E**) FBXW7-mediated K48 ubiquitination of p53 leads to its degradation and promotes cell cycle recovery. (**F**) Upon DNA damage, phosphorylated SREBP1 is ubiquitinated by FBXW7, and its degradation results in reduced expression of lipogenic enzymes. (**G**) Peli1 ubiquitinates NBS1, a component of the MRN complex, which facilitates ATM recruitment to a DSB site and promotes HR. (**H**) In CD8^+^ TIL, Peli1 ubiquitinates mTORC1 inhibitor TSC1 at K63, resulting in downregulation of metabolic reprogramming and effector functions of CD8^+^ T cells.

Neural precursor cell expressed developmentally downregulated protein 4 (NEDD4, also known as NEDD4-1) is a HECT type E3 ligase that mediates ubiquitination and proteasomal degradation and regulates the cellular localization and stability of substrates, mainly membrane receptors (e.g., insulin-like growth factor-1 receptor (IGF-1R), vascular endothelial growth factor receptor-2 (VEGFR2), and epidermal growth factor receptor (EGFR)), as well as proteins related to tumorigenesis (e.g., Mdm2, Beclin1, p21, and PTEN) [121,122]. NEDD4 participates in and regulates the DNA damage response (DDR) to preserve genome integrity. RNA polymerase II (RNAPII), which is crucial for the synthesis of mRNA and transcripts following DNA repair, becomes stalled at DNA damage sites and is degraded during DDR [123]. This process is tightly regulated, and its regulation is crucial for proper response to DNA damage-induced stress [123]. The ubiquitination of RNAPII after UV-induced DNA damage is mediated by NEDD4, and it is eventually degraded by the proteasome [124] (Figure 2A). Furthermore, Mdm2, a negative regulator for tumor suppressive protein p53, is also a target for NEDD4 [125]. The p53 pathway produces a set of proteins that are directly involved in DNA repair [126], and NEDD4 regulates Mdm2 stability by K63-linked ubiquitination and, in doing so, affects p53 signaling, thus contributing to the DNA damage repair process [125]. Together, these studies suggest NEDD4 as a regulator of both DNA damage sensing and the subsequent response.

NEDD4 is well known to initiate and promote cancer as it degrades tumor suppressor protein PTEN through poly-ubiquitination and dictates nuclear localization via mono-ubiquitination [127,128]. These roles indicate a central role in tumorigenesis. PTEN also plays a crucial role as a regulator that participates in the metabolism of glucose, lipid, and mitochondria through modulation of PI3K/AKT pathways which are commonly activated in cancer [129,130]. Considering that loss of PTEN results in lower insulin resistance [131] and lipogenesis [132], which is responsible for tumor microenvironment establishment [133], NEDD4 reprograms metabolic status and tumor progression through PTEN ubiquitination (Figure 2B). Moreover, AKT, a major effector enzyme that promotes metabolic reprogramming in cancer cells [134,135], is also a target of NEDD4 [136,137]. In response to insulin and IGF-1, NEDD4 promotes K63-linked ubiquitination of membrane-bound AKT, independent of phosphorylation status, and regulates its nucleus-orientated trafficking [136]. Additionally, NEDD4 more efficiently recognizes a cancer-derived plasma membrane-philic mutant AKT (E17K) and regulates its trafficking into the nucleus, thus suggesting an oncogenic role of NEDD4 by AKT ubiquitination [136]. NEDD4 also promotes ubiquitination and degradation of Beclin 1, a tumor-suppressive protein and a central autophagy mediator. Now that the crosstalk between autophagy and cancer metabolism is more fully appreciated, it can be assumed that NEDD4-mediated regulation of Beclin1 affects tumor progression by reprogramming cancer metabolism [138]. In contrast, low NEDD4 levels lead to worse outcomes in multiple myeloma (MM) patients. NEDD4 induces p-AKT K48-linked ubiquitination, resulting in its degradation, and depletion of NEDD4 in MM cells resulted in decreased drug sensitivity by elevating p-AKT, thereby indicating that NEDD4 may also have a tumor suppressive role [137]. The involvement of NEDD4 in DNA damage and cancer metabolism makes it a promising target for cancer therapy. The role of NEDD4 in oncogenesis is not yet fully clarified, and further investigation into DNA damage and cancer metabolism may lead to a greater focus on NEDD4 as a novel target for cancer therapy.

### 4.2. APC/C^CDH1^

APC/C^CDH1^ is an E3 ligase that plays a central role in the cell cycle, especially at the G1 stage, and its tight regulation is crucial to preventing the development of diseases and cancer [139]. Since cell cycle progression is involved in both DNA damage repair and metabolic activities, APC/C^CDH1^ plays a role as a bridge between them [140,141,142,143]. DNA damage can interrupt the normal progression of the cell cycle, leading to cell death or carcinogenesis [144]. APC/C^CDH1^ is activated during cell cycle arrest at the G2 stage to allow time for DNA repair in response to DNA damage and directly interact with DDR-related proteins [142]. E2F1, a transcription factor that regulates cell cycle progression, DNA replication, and DDR, is controlled by APC/C^CDH1^-induced K11 linkage-specific ubiquitination [145]. APC/C^CDH1^ ubiquitinates E2F1 for proteasomal degradation, while it is blocked in response to DNA damage reagent treatment [145] (Figure 2C). Considering that appropriate downregulation of E2F1 is critical to maintaining genomic stability, blocking this process allows the inhibition of cancer cell growth, thus suggesting a critical role of APC/C^CDH1^ in DNA damage and cancer. It has also been reported that there is a direct relationship between DDR and APC/C^CDH1^. Specifically, deletion of CDH1 has been found to result in increased DNA damage and sensitized DNA double-strand break-inducing agent response by targeting CtBP (C-terminal binding protein) interacting protein (CtIP), a protein that plays a role in DNA double-strand break (DSB) repair [146]. APC/C^CDH1^ induces ubiquitination of CtIP, regulating its stability, both in the normal progression of the cell cycle after mitotic exit and in response to DNA damage during the G2 phase [146]. Regulation of CtIP levels plays a crucial role in proper DNA repair in response to damage, either through the NHEJ pathway or HR [146,147]. The interaction between APC/C^CDH1^ and CtIP ensures appropriate DNA repair in response to damage [146], making APC/C^CDH1^ a critical regulator in the DDR mechanism.

In cancer cells, glucose metabolism is often modulated by altering the level and activity of proteins involved in glycolysis for adaptation to the cancer microenvironment, a phenomenon known as the Warburg effect [148]. APC/C^CDH1^ is involved in this process by promoting ubiquitination and degradation of 6-phosphofructo-2-kinase/fructose-2,6-bisphosphatase, isoform 3 (PFKFB3), a glycolysis-promoting enzyme (Figure 2D). In several types of cancer, including breast, colon, and lung cancer, PFKFB3 is overexpressed, and it leads to an increase in the levels of fructose-2,6-bisphosphate, an enzyme that promotes cell proliferation and survival, and thus contributes to cancer cell growth and proliferation [149]. Since APC/C^CDH1^ negatively regulates the level of PFKFB3 in cancer cells by ubiquitination, APC/C^CDH1^ plays a role in inhibiting cancer cell growth [150,151]. Therefore, when CDH1 is depleted, increased PFKFB3 causes an upregulation of glycolysis and cell proliferation, suggesting that APC/C^CDH1^ modulation is important in cancer metabolism alteration [150]. Additionally, APC/C^CDH1^ takes isocitrate dehydrogenase 3β (IDH3β), an enzyme involved in the TCA cycle, as a substrate to affect glycolysis and the level of PFKFB3 [152]. APC/C^CDH1^ promotes ubiquitination and degradation of IDH3β, of which overexpression leads to altered metabolism and enhanced PFKFB3 levels that are related to poor outcomes in ESCC [152] (Figure 2D).

In addition to its role in regulating glucose metabolism, APC/C^CDH1^ also plays a role in amino acid metabolism as it regulates phenylalanine hydroxylase (PAH), a key enzyme in the metabolism of phenylalanine, and glutaminase (GLS) ubiquitination [153,154]. In HCC, the expression of APC/C^CDH1^ and PAH is strongly correlated with good clinical outcomes as APC/C^CDH1^ promotes poly ubiquitination of PAH for reduced stability and degradation by 26S proteasome [153]. APC/C^CDH1^ also controls the activity of GLS by targeting it for ubiquitination and degradation [154]. This process helps to regulate the levels of GLS and thus regulates glutamine metabolism, which is vital for maintaining normal cellular processes such as cell growth, proliferation, and survival [154]. In colorectal cancer, GLS is overexpressed, and increased interaction of APC/C^CDH1^ and GLS by selenite treatment induces degradation of GLS that appears to contribute to inhibition of cancer progression [154].

Although studies have advanced our understanding of the role of APC/C^CDH1^ in metabolic regulation, DDR, and DNA repair, there is still much that is not known about its specific mechanisms and interactions with other proteins and pathways. As cell cycle regulation is involved in both metabolism and DDR, a direct role for APC/C^CDH1^ as a link between them is worth elucidating. Therefore, further studies are needed to gain a full understanding of the function of APC/C^CDH1^ as a nexus in these processes.

### 4.3. FBXW7

FBXW7 (also known as FBW7) is an F-box protein that preferentially recognizes phosphorylated substrate [155]. It is a component of the Skp2-Cullin-F-box (SCF) E3 ligase, which ubiquitinates target proteins to regulate cellular processes. The FBXW7 gene is a tumor suppressor gene that undergoes mutation or deletion in a variety of human cancers, including colon, liver, lung, and ovarian cancer [155]. FBXW7 takes various DDR proteins as substrates, such as p53 [156,157,158], p63 [159], polo-like kinase 1 (PLK1) [160], bloom helicase (BLM) [161], SRY-box transcription factor 9 (SOX9) [162], and X-ray repair cross-complementing 1 (XRCC4) [163], highlighting its role in DDR. Specifically, FBXW7 not only contributes to K48-linked polyubiquitination and degradation of p53 in the normal state but also during and after DNA damage upon exposure to UV, DSB inducers, and ionizing radiation (IR) [156,157,158] (Figure 2E). When DNA damage occurs, FBXW7 and p53 form a negative feedback loop for tight regulation, in which increased p53 levels after DNA damage trigger the expression of FBXW7 and its enhanced binding to phosphorylated p53 by ATM, resulting in the degradation of p53 and contributing to resumptions in the cell cycle after DNA repair [156,157,158]. When DNA damage occurs, FBXW7 also promotes ubiquitination of p63, a p53-related protein that has similar functions to p53 [159]. Furthermore, FBXW7 induces K48-linked ubiquitination on PLK1 and BLM for subsequent degradation [160,161]. PLK1 promotes DNA replication, and its degradation by FBXW7 occurs in response to UV-induced DNA damage that blocks the formation of pre-replicative complexes, preventing the spread of cells with damaged DNA. FBXW7, therefore, acts as a gatekeeper for genome stability [160]. BLM, a 3′−5′ ATP-dependent RecQ DNA helicase that stabilizes DNA replication, the NHEJ pathway, and HR of DSB, is recruited to the DNA damage site to fix the errors [164]. During mitosis, FBXW7 promotes ubiquitination and degradation of BLM by a glycogen synthase kinase β (GSK3β) and CDK2/Cyclin A2-dependent phosphorylation on Thr171 and Ser175, which requires prior phosphorylation on Thr182 by Chk1/Chk2 [161]. Direct evidence that links FBXW7 and BLM to DDR has not yet been established. However, considering Chk1/Chk2 is activated during DDR [165], FBXW7-mediated degradation is likely to be involved in DDR. Furthermore, SOX9 ubiquitination and degradation are catalyzed by FBXW7 [162]. In response to chemotherapeutic drugs and UV irradiation, FBXW7 induces ubiquitination and degradation of SOX9 in a GSK3β-dependent manner. As overexpression of SOX9 leads to increased survival after UV irradiation, the involvement of FBXW7 in degrading SOX9 in response to DNA damage is crucial to prevent cells from becoming malignant [162]. FBXW7 also induces K63-linked polyubiquitination for protein activation. In response to IR, FBXW7 is recruited to DSB sites in an ATM-dependent manner to interact with phosphorylated XRCC4 [163]. FBXW7 promotes K63-linked polyubiquitination of XRCC4, which results in increased survival after IR-induced DNA damage through enhanced NHEJ complex formation facilitating interaction with Ku70/80, XRCC4 localization to DSBs, and effective NHEJ repair [163].

FBXW7 also contributes to its tumor suppressive role through modulation of metabolic activities as it is known to induce ubiquitination and degradation of sterol regulatory element binding protein 1 (SREBP1), a transcription factor that regulates lipid metabolism [42,166] (Figure 2F). The loss of FBXW7 leads to stabilization and accumulation of SREBP1, which in turn triggers activation of AKT and enhances expression of target gene sets involved in cholesterol metabolism, thereby supporting cancer cell survival and proliferation [166]. In addition, in breast cancer cell lines and primary tumors, the mammalian target of rapamycin (mTOR), a master regulator of metabolism that balances anabolism and catabolism, is targeted by FBXW7 for ubiquitination and degradation [167]. The deletion of FBXW7 results in the loss of mTOR, p-mTOR, and as well as phosphorylated S6-kinase (p-SK6), a known mTOR downstream target [167]. FBXW7 is also known to induce K48-linked ubiquitination of c-Myc, a transcription factor that regulates the transcription of genes related to metabolism [168,169]. BLM-induced phosphorylated c-Myc shows enhanced interaction with FBXW7 leading to altered transcription of metabolic genes (e.g., EGFR, PIM1, and FGF9) and thus initiation of c-Myc driven tumors in a xenograft mouse model [169]. Moreover, FBXW7 can target hypoxia-inducible factor-1α (HIF-1α) for ubiquitin-dependent degradation during hypoxia, which causes metabolic changes and angiogenesis in cancer cells [170,171]. FBXW7 recognizes hypoxia-induced phosphorylated HIF-1α for degradation via GSK3β, thereby the loss of FBXW7 or GSK3β results in increased hypoxia-induced stimulation of angiogenesis and contributes to tumor growth [170,171]. FBXW7-mediated p53 degradation can also affect metabolic change by altering the expression of genes involved in cellular metabolism, such as glycolysis, lipogenesis, and oxidative phosphorylation, all of which are relevant to the development of cancer [172].

Current studies focus on the role of FBXW7 as a bridge between DDR and cancer metabolic reprogramming. Notably, in one recent study, FBXW7 deficiency led to defective DNA damage repair and altered metabolic features, which increased NADPH consumption with enhanced sensitivity to radiotherapy in IDH1 mutant cancer cells [42]. In addition to this outcome, FBXW7 is responsive to DNA damage, and its target proteins often regulate metabolism as well as DDR. As a connector of DDR and metabolism, targeting FBXW7 makes a lot of sense as a therapeutic intervention for targeting cancer, tuning DDR and metabolic programs at the same time.

### 4.4. Pellino1

Pellino1 (Peli1) is a receptor signal-responsive RING-like type E3 ubiquitin ligase that preferentially recognizes and targets phosphorylated proteins [173,174]. Peli1 participates in innate and adaptive immune responses and is activated upon various receptor signals such as T cell receptor (TCR), B cell receptor (BCR), and toll-like receptors (TLRs) [173,175,176,177,178,179]. In response to these signals, Peli1 plays a key role as a modifier of the downstream signaling cascade regulating immune cell proliferation, activation, and differentiation [175,180], as well as regulating stress signals (e.g., inflammation, oxidative stress, and ER stress) [181,182,183], and promoting autoimmune disease and cancers [175,176,180,183,184,185].

In the last few years, the importance of ubiquitination in DSB response and repair has been investigated, as dysfunctions of ubiquitin signaling factors in DSB repair are relevant to immune disorders and carcinogenesis [6,7,186,187,188,189,190]. Intriguingly, a recent study revealed that Peli1 is also responsive to DSB. Peli1 includes forkhead-associated (FHA) domains, which are a prevalent structure in enzymes involved in DDR [77,191,192]. Indeed, Peli1 is recruited to DSB sites as an early response protein mediated by the FHA domain. When DSB occurs, the master kinase ATM activates Peli1 by phosphorylation to recruit it to the DSB site. Then accumulated Peli1 activates ATM through K63-linked ubiquitination of NBS1 in ATM- and γH2AX- dependent manners and contributes to overall DNA damage sensing, signaling, and repair process; in particular DNA-end resection-mediated HR repair [77] (Figure 2G). Additionally, Peli1 in microglia negatively regulates TLR-mediated type Ι IFN induction, which is known to be regulated by DNA damage and ATM via the STING pathway [193,194]. Considering that Peli1 is crucial in both DNA repair and induction of type Ι IFN, Peli1 may play a role as a regulator between type Ι IFN induction and DDR or ATM. As well, Peli1 induces K63-mediated Bcl6 ubiquitination, which promotes B cell lymphomagenesis. In mice, overexpression of Peli1 stabilizes Bcl6 resulting in B cell lymphomagenesis, and in diffuse large B cell lymphoma (DLBCL) patients, poor prognosis is followed by a higher level of Peli1 and Bcl6, indicating that Peli1 is a novel oncogenic signal in B cell lymphoma [175]. Now that the role of Peli1 in DDR is known and as Bcl6 sustains the lymphoma phenotype and enables tumor cells to be survived by modulating the DNA damage repair process [195,196], the underlying mechanism of Peli1 to deteriorate B cell lymphoma by activating Bcl6 is possibly associated with DNA damage repair process.

A growing body of evidence suggests the link between DNA damage and cancer metabolism, which can be exploited therapeutically. Interestingly, the role of Peli1 in cancer and cancer metabolism has been well established. The overexpression of Peli1 induces the development of tumors in various organs, such as the liver, lung, thymus, and spleen, and the formation of tumors results in a shorter lifespan in vivo [175]. In humans, the expression of Peli1 is upregulated in lymphoid and several solid cancers and is correlated with poor prognosis, indicating the pro-tumorigenic role of Peli1 [175,185,197,198]. Recently, the crucial role of Peli1 in regulating phosphatidylinositol-3-kinase (PI3K)-AKT signaling, the major effector pathway that reprograms cellular metabolism in cancer, has been revealed [185,199]. The inhibition of Peli1 in papillary thyroid cancer (PTC) cells induces downregulation of the PI3K-AKT pathway and leads to suppression of tumor growth, proliferation, and migration [185].

In TME, it is not only tumor cells but also infiltrated immune cells in the TME that undergoes metabolic reprogramming, and this intimately affects the malignant progression of tumors [200]. In particular, tumor cells dampen the antitumor immune responses of CD8^+^ T cells by altering their metabolism to evade immune surveillance [201]. Recent studies have demonstrated that Peli1 regulates the metabolism of T cells to attenuate their cytotoxic role and antitumor functions. The expression of Peli1 is upregulated among tumor-infiltrating CD8^+^ T cells, and this seems to change the metabolism of tumor-infiltrating CD8^+^ T cells within the TME by inhibiting TCR signal transduction, resulting in suppressed survival, proliferation, and effector functions with reduced GzmB and IFNγ expression [184,202]. Accordingly, Peli1 deficiency in T cells leads to a reduction in exhausted tumor infiltrating CD8^+^ T cells while enhancing their effector functions, thereby establishing antitumor immunity [184]. Furthermore, Peli1 suppresses the activation of the metabolic kinase, mTORC1 by promoting TSC1-TSC2 dimerization in T cells (Figure 2H). Hence, Peli1-deficient T cells exhibit high metabolic activities, especially glycolysis, resulting in decreased tumor growth, and increased tumor-infiltrating T cells with stronger antitumor function [180].

Given that Peli1 expression is exceedingly suppressed under non-pathological conditions, while it is activated and upregulated in diseases such as cancer [175,176,183,185,197], it is widely accepted that regulating Peli1 could be a highly advantageous strategy in cancer treatments. Considering the impact of deregulated DNA damage repair in cancer development and progression [144], the novel role of Peli1 in regulating DNA damage repair becomes more remarkable. Moreover, since modulating DNA repair is considered an efficient strategy to sensitize cancer cells to radiotherapy [203,204], the regulation of Peli1 expression or activity may be an attractive therapeutic approach. Growing evidence indicates that cancer metabolism and DNA damage are intrinsically linked, and targeting these pathways is likely to be a productive new therapeutic strategy for cancer [8]. As recent studies underline that Peli1 also interplays between cancer metabolism and DNA damage, the exact roles of Peli1 in metabolic reprogramming, DNA repair process, and their link are worth further studied and analyzed as an effective therapeutic target for cancers.

## 5. Current Therapeutic Implications in Cancer Targeting Ubiquitination

As abnormalities in ubiquitination are highly relevant to the pathogenesis and malignance of cells, small molecule inhibitors that inhibit ubiquitin signaling are now being tested. Now that the role of ubiquitin ligase as a linker between DNA damage and cancer metabolism is turned out to be crucial in cancer development, targeting ubiquitination and related enzymes is regarded as a more efficient strategy to cure cancer. To modulate ubiquitination in cancer, targeting UPS, including the proteasome, E1 enzymes, E2 enzymes, E3 ligases, and deubiquitinases (DUBs), is suggested as a therapeutic approach [205]. Currently, the small molecule inhibitors that target the different components of the UPS offer promising therapeutic agents to combat cancer, and indeed, enormous efforts have been made to find drugs targeting UPS (Table 3).

Proteasome inhibitors (PIs) such as bortezomib, carfilzomib, oprozomib, and ixazomib have shown successful outcomes [206]. For instance, the first-in-class PI drug, bortezomib, reversibly targets the 20S proteasome and inhibits and reduces its chymotrypsin-like, trypsin-like, and caspase-like activities [206]. In several cancer cell lines, including multiple myeloma (MM) and mantle cell lymphoma (MCL), treatment with bortezomib resulted in NF-κB pathway inhibition, cleavage of Mcl-1 and prompted c-Jun/AP-1 pathway [207,208]. Bortezomib is now used clinically as an anti-cancer drug. However, since the PIs act in the last step of the ubiquitin–proteasome system (UPS), the cause is not radically solved in some cases. Hence, there are notable adverse effects, including bortezomib-induced peripheral neuropathy (BIPN), which is associated with the accumulation of Ub-laden proteins [209]. Consequently, drugs targeting other UPS components have been developed.

One of these targets the E1 enzyme, which is responsible for ubiquitin activation. TAK-243 (also known as MLN7243) inhibits the ubiquitin-activating enzyme (UAE) by forming a TAK-243-ubiquitin adduct [210]. Its treatment resulted in disruptions to the cell cycle, altered DNA damage repair, and suppression of cancer cells. In the xenograft mice model, TAK-243 inhibits tumor growth driven through UAE-specific antitumor activity [210]. A clinical trial for TAK-243 in a solid tumor was terminated in phase 1 (NCT02045095), establishing the maximum tolerated dose (MTD), and a subsequent study (NCT03816319) is now enrolling patients with leukemia.

E2 enzymes which conjugate ubiquitins to substrates are also being explored as targets. Inhibitors suppress E2 enzymes by small molecules, by miRNA, or by preventing the interaction of E2s with E1s and E3s [211]. In past decades, CC0651, a small molecule selective allosteric inhibitor of E2 enzyme Cdc34, was investigated [212]. The treatment of CC0651 promotes p27 and cyclin E substrates stabilization leading to decreased cell proliferation in human cancer cell lines suggesting CC0651 is a candidate for anti-cancer therapy [212]. NSC697923 inhibits UBE2N (also known as Ubc13), a regulator of p53, to suppress nuclear tetramerization [213,214]. In a neuroblastoma cell line, NSC697923 promotes p53 nuclear localization and JNK pathway activation, inducing apoptosis of the cell [214]. In vivo, NSC697923-treated xenograft mice show decreased tumor growth suggesting NSC697923 may serve as a potential drug for cancer [214]. Leucettamol A was found to interfere with the interaction between E1s and E2, specifically UBC13 and UEV1A [215]. In addition, Manadosterols A and B share the same target as Leucettamol A [216]. E2 enzyme inhibition has also been investigated through miRNAs. For instance, miR661-3p and miR-381-3p that target E2 enzymes are being investigated [217,218]. Although these miRNAs showed efficacy in vitro and/or in vivo, miRNAs have limitations, such as instability and unexpected side effects when applied to multiple targets [219]. To overcome these limitations, various types of delivery systems, pre-toxicity testing before clinical trials, and strategies to express miRNA in specific tissues or organs should be elucidated.

E3 ligase is the most crucial component in the ubiquitination pathway that recognizes substrates with high specificity and has been widely studied to cure cancer [220]. For instance, Nutlins are chemicals that inhibit the interaction between Mdm2 and tumor suppressor p53, which allows cells to restore their ability to suppress cell cycle progression and induce apoptosis [221,222]. In clinical trials, Nutlin-3a (also known as idasanutlin) has completed phase 1 trials (NCT03362723, NCT02828930, NCT02670044), and now recruitment is progressing for phase 1/2 (NCT04029688) in solid tumors and leukemia. Moreover, other Mdm2 inhibitors and combination therapy with other drugs are now in clinical trials. These include KRT-232 (AMG 232; NCT02110355, NCT01723020, NCT03031730), Milademetan (DS-3032b; NCT01877382, NCT03671564), and HDM201 (NCT05180695, NCT03714958, NCT03940352, NCT02343172), which are in phase 1 or 2, indicating that targeting Mdm2 to reactivate p53 is clinically promising. In addition, ALRN-6924, a dual inhibitor for Mdm2 and Mdmx, is now in phase 1 clinical trial for solid tumors (NCT03725436) [223]. Furthermore, antagonists of the E3 ligase IAP family that targets its BIR domains are also suggested to activate noncanonical NF-kB signaling and promote TNF-mediated cell death [224]. IAP inhibitor GDC-0917 (NCT01226277) has completed phase 1 in solid tumor and lymphoma, while Debio1143 (Xevinapant) has completed phase 1 or 2 (NCT01078649, NCT03871959, NCT04122625) and is now enrolling phase 3 (NCT04459715, NCT05386550) in neck and head tumors. Promising results in clinical trials and the involvement of the IAP family in metabolism and DDR has unraveled, targeting the IAP family in cancer metabolism and DDR is worth further study. Another E3 ligase to be targeted for cancer therapy is the APC/C, which regulates DDR and cell cycle progression. APC/C combines with substrate adaptor CDC20 or CDH1 for their activation [225]. Therefore, inhibiting the interaction between APC/C and its adaptor is a strategy to inhibit APC/C ligases. Apcin inhibits the interaction of APC/C with substrate adaptor CDC20, and in osteosarcoma cells, treatment of apcin-inhibited cell growth and induced apoptosis [226]. Tosyl-l-arginine methyl ester (TAME), which binds to the APC3 subunit, interrupts APC/C interaction with both adaptor proteins, CDC20 and CDH1 [227]. In glioblastoma (GBM) cell lines, a combination treatment of TAME and apcin effectively decrease cell viability and induces mitotic arrest, indicating that drugs for targeting APC/C are promising chemotherapeutics [228]. As noted, the SCF family, which includes FBXW7, SKP2, and β-TrCP, affects various proteins that regulate overall cancer development, which makes it an attractive target to combat cancer. As FBXW7 plays a role as a cancer suppressor, an agonist of the FBWX7 E3 ligase complex, oridonin degrades c-Myc and induces apoptosis in leukemia and lymphoma cells [229]. Various drugs, including SZL-P1-41, Longikaurin A (LK-A), curcumin, and dioscin, inhibit SKP2. In vitro, SZL-P1–41 inhibits the interaction between SKP1 and SKP2 and consequently restricts cancer cell survival in a p53-dependent manner, along with inhibited aerobic glycolysis and enhanced anti-tumor activity in vivo [230]. LK-A and curcumin inhibit the expression of SKP2 [231,232]. In hepatocellular carcinoma cells, LK-A inhibits cell proliferation and induces cell cycle arrest and apoptosis through JNK/c-Jun pathway activation by ROS accumulation, and the suppression of tumor growth was confirmed in a xenograft model [231]. In pancreatic cancer cells, treatment with curcumin also showed similar results to LK-A [232]. Clinical trial for curcumin in cancer is now actively progressing and some are recruiting for phase 3 (NCT03769766, NCT02064673) in prostate cancer. Dioscin is a novel inhibitor of SKP2 that promotes ubiquitin-dependent degradation of SKP2 that leads to suppressed tumor growth in vivo [233]. For β-TrCP, GS143 and erioflorin were identified as inhibitors. GS143 targets β-TrCP by inhibiting interaction with IκB that leads to markedly decreased ubiquitination and degradation of IκB, blocking the NF-κB signaling pathway [234]. Erioflorin interferes with the interaction of β-TrCP with programmed cell death 4 (Pdcd4), a tumor suppressor, resulting in stabilization of Pdcd4, reducing AP-1- and NF-κB-dependent transcription that alters the cell cycle and inhibits proliferation in vitro [235].

DUB is also considered a therapeutic target as it is directly and indirectly associated with cancer. For instance, the ubiquitin-specific protease (USP) 14, which prevents substrate degradation, is overexpressed in several cancers, and its expression positively correlates with poor prognosis [236]. The USP14 inhibitor, IU1, abrogates its enzymatic activity and lowers the tumor recurrence rate, and improves prognosis [236]. Consequently, treatment with IU1 analog, IU1-47, prevents proliferation and induces autophagic cell death in lung cancer cells [237]. Another small molecule inhibitor, WP1130 can inhibit USP14 as well as USP9x, USP5, UCH37, and UCHL1, which regulates cell survival [238]. In cancer cell lines, DUBs inhibition by WP1130 induces apoptosis without changing proteasome activity [238,239]. USP7 is also considered a potential target for cancer therapy, and recent studies have found various UPS7 inhibitors that stabilize MDM2 and contribute to decreased tumor suppressor p53 indirectly. After the finding of HBX 41,108, which induces p53-dependent apoptosis, many other small molecules have been shown to target USP7, although there are a few remaining challenges to overcome, such as poor selectivity [240,241].

## 6. Conclusions

In this review, we highlight the role of ubiquitination on cancer metabolism and genetic integrity. Ubiquitination regulates DNA integrity and metabolism by controlling substrates that affect both functions or by regulating the substrates that specifically act on either DNA integrity or metabolism. This interconnected regulation is critical for maintaining overall cellular homeostasis and ensuring that any changes in DNA integrity or metabolism are closely monitored and coordinated for optimal cellular function. Especially in cancers characterized by metabolic reprogramming and aberrant DNA repair functions, co-regulation of DNA integrity and metabolism through ubiquitination may further increase its potential as a therapeutic target for cancer treatment. A comprehensive understanding of the role of ubiquitination in regulating DNA integrity and metabolism in cancer can provide valuable insights into potential therapeutic targets and underlying mechanisms for the development of effective cancer therapies.

## Figures and Tables

**Table 1:** E3 Ubiquitin ligase in cancer metabolism.

Name	Type	Substrate	Ubiquitination Residue	Cancer Type	Ref. (PMID Number)
E4F1	atypical	p53	K48	osteosarcoma, hepatocellular carcinoma	17110336, 24163401
HectH9	HECT	c-Myc	K63	retinoblastoma	31677785
HUWE1	HECT	HAUSP	K63	lung cancer, prostatic adenocarcinoma	30176860, 27934968
		c-Myc	K48, K63	breast cancer	30176860
		p53	K48	B cell lymphoma	30176860
		WIPI2	unknown	pancreatic cancer	30340022, 34502089
		HK2	K63	prostate cancer	31201299
NEDD4	HECT	AKT	K63, K48	breast cancer, hepatocellular carcinoma	23195959, 31390487
		PTEN	K63	prostate cancer	17218261, 17218260
		Beclin-1	K11	adenocarcinoma	21936852
NEDD4L	HECT	ULK1	K27, K29	adenocarcinoma	27932573
UBR5	HECT	citrate synthase (CS)	unknown	breast cancer	30850587
WWP1	HECT	PTEN	K27	prostate cancer	31097636
WWP2	HECT	PTEN	unknown	prostate cancer	21532586
PARKIN	RBR	mTOR	unknown	ganglioglioma	28803490
		HIF-1α	unknown	breast cancer	29180628
		PKM2	unknown	lung cancer, glioblastoma	26975375
		PHGDH	unknown	breast cancer, lung cancer	32478681
APC/C^CDH1^	multimeric RING	PFKFB3	unknown	neuroblastoma, adenocarcinoma	20080744, 24604252, 21402913
		PAH	K48, K63 (no direct evidence)	hepatocellular carcinoma	33260674
		GLS1	unknown	colorectal cancer	27902968
		IDH3β	unknown	esophageal squamous cell carcinoma	31053633
BRCA1	RING	AKT	K48	breast cancer	19074868
CARP1/2	RING	p53	K48	lung cancer, colorectal cancer	17121812
COP1	RING	p53	K48	osteosarcoma	15103385
		FASN	unknown	adenocarcinoma	23269672
CRL4A	RING	AMPKα	K29, K63	ovarian cancer	30807229
CUL3-KLHL20	RING	ULK1	K48	adenocarcinoma	26687681
CUL3-KLHL22	RING	DEPDC5	K48	breast cancer	29769719
CUL3-KLHL25	RING	ACLY	unknown	lung cancer	27664236
CUL3-SPOP	RING	PrLZ	K48	prostate cancer	35194188
CUL4-DDB1	RING	Raptor	K63	lung cancer	18235224
CUL4-DDB1 (AMBRA1)	RING	Beclin-1	K63	colon cancer, pancreatic ductal adenocacinoma	27308402, 23974797
CUL5	RING	p53	K48	B-cell chronic lymphocytic leukemia	17449237, 32194910
CUL7	RING	p53	unknown	lung cancer, breast cancer	17942889, 25003318
GID	RING	AMPK	K48	adenocarcinoma, osteosarcoma	31795790, 25679763
Gp78	RING	HMGCR	unknown	breast cancer, prostate cancer	22143767, 33718197
MAGE-TRIM28	RING	FBP1	unknown	hepatocellular carcinoma	28394358
MDM2	RING	p53	K48	breast cancer	9153396, 34650049
MDM2	RING	PGAM	unknown	lung cancer	24567357
MKRN1	RING	p53	K48	lung cancer, colon cancer	19536131, 20930521
MULAN	RING	AKT	K48	adenocarcinoma	22410793
Pirh2	RING	p53	K48	osteosarcoma	12654245
RFP	RING	PTEN	K48	plasmacytoma, myeloma, endometrial cancer, colon carcinoma	23419514
RING1	RING	p53	K48	liver cancer	29187402
RNF2	RING	AMBRA1	K48	thymus lymphoma	24980959, 28789945
RNF5	RING	SLC1A5/ SLC38A2	unknown	breast cancer	25759021
RNF8	RING	histone H3	K48	breast cancer, epidermoid carcinoma	28507061
RNF115	RING	p53	unknown	lung adenocarcinoma	32553631
RNF126	RING	mTOR	K48	acute myeloid leukemia	32131492
		PDK	unknown	breast cancer	27462466
RNF139	RING	SREBP1 precursor	unknown	renal cell carcinoma	20068067
RNF145	RING	HMGCR	unknown	adenocarcinoma, hepatocellular carcinoma	29374057, 30543180
RNF152	RING	RagA	K63	non-small cell lung cancer	25936802, 32486221
		Rheb	unknown	colon cancer	30514904, 32486221
RNF216	RING	Beclin-1	K48	adenocarcinoma	29361549
SCF^FBXL6^	RING	HSP90AA1	K63	hepatocellular carcinoma	32576198
SCF^FBXL8^	RING	mTOR	K48	colorectal carcinoma	27916606
SCF^FBXL14^	RING	c-Myc	K48?	glioma	27923907
SCF^FBXL18^	RING	AKT	K63	glioma	27926990
SCF^FBXL20^	RING	VPS34	unknown	adenocarcinoma, glioma	25593308
SCF^FBXO32^	RING	c-Myc	K48	ovary cancer	25944903
SCF^FBXW7^	RING	SREBP1	unknown	glioma	34737211, 36176395
		mTOR	K48	breast cancer	30086763, 18787170
		HIF-1α	K48	ovarian cancer	21964756
		c-Myc	K48	T-acute lymphoblastic leukemia, uterine cancer, colorectal cancer, bladder cancer, lung cancer	30086763, 33260160, 23750012, 20852628, 28665315
		p53	K48	adenocarcinoma, colorectal cancer	31905981, 25314076
SCF^SKP2^	RING	c-Myc	K48	B cell lymphoma	12769844, 12769843, 28665315
		AKT	K63	breast cancer	22632973
		RagA	K63	adenocarcinoma	26051179
SCF^β-TrCP^	RING	DEPTOR	unknown	breast cancer, ovarian cancer	22017876, 22454292
		Myc	K33, K48, K63	osteosarcoma	20852628
		PFKFB3	unknown	adenocarcinoma	21402913
		NRF2	unknown	endometrioid carcinoma	25937177
SIAH2	RING	α-KGDHC	unknown	adenocarcinoma, tongue cancer, renal cancer	15466852, 24506869
Synoviolin	RING	p53	K48	colon cancer	25128494, 20930521, 17170702
TOPORS	RING	HIF-1α	K63	colon cancer	23722539
TRAF2	RING	mLST8	K63	ovarian cancer, melanoma, adenocarcinoma	28489822
TRAF4	RING	AKT	K63	lung cancer	24154876
TRAF6	RING	p62	K63	prostate cancer, lung cancer	23911927
		AKT	K63	prostate cancer	19713527
		HIF-1α	K63	colon cancer, adenocarcinoma	23722539
		ULK1	K63	chronic myeloid leukemia	30929559
		Beclin-1	K63	leukemia	20501938
		HK2	K63	liver cancer	28980855
TRC8/RNF139	RING	HMGCR	unknown	renal cell carcinoma	20068067
TRIM16	RING	NRF2	K63	adenocarcinoma	30525100
		ULK1	K63	acute monocytic leukemia	27693506
TRIM21	RING	PFKP	K48	glioma	29038421
TRIM25	RING	PTEN	K63	lung cancer	33931764
TRIM31	RING	TSC1-TSC2	K48	hepatocellular carcinoma	28967907
		p53	K48	breast cancer	34650049
TRIM32	RING	ULK1	K63	colon cancer, lung cancer, hepatocellular carcinoma	31123703
TRIM45	RING	p53	K48	glioma	28542145
TRIM50	RING	Beclin-1	K63	adenocarcinoma	29604308
TTC3	RING	AKT	K48	B Cell Lymphoma	20059950
VHL	RING	HIF-1α	K48	adenocarcinoma	25958982, 12086861
XIAP	RING	HIF-1α	K63	osteosarcoma, renal cell carcinoma	28666324
ZNRF1	RING	AKT	K48	neuroblastoma	22057101
Peli1	RING-like	PKC theta	K48	T cell lymphoma, colon adenocarcinoma	35058288
		TSC1	K63	melanoma	33215753
CHIP	U-box	AKT	K48	breast cancer, adenocarcinoma	21767636
		p53	K48	lung cancer, colon cancer	29953728

**Table 2:** E3 Ubiquitin ligase in DNA damage.

Name	Type	Substrate	Ubiquitination Residue	Ref. (PMID Number)
ARF-BP1/Mule	HECT	p53	unknown	15989956
		MCL-1	unknown	15989957
E6-AP	HECT	p53	unknown	31749782
HUWE1	HECT	histone H1	unknown	29127375
ITCH	HECT	p73	unknown	15678106
		p63	unknown	16908849
		WWOX	K63	25331887
		H1.2	K48, K63	30517763
NEDD4	HECT	RNA PolⅡ	unknown	17996703
		Mdm2	K63	24413081
NEDD4L	HECT	OGG1	unknown	33282879
Rsp5	HECT	RNA PolⅡ	unknown	9108033
Smurf1	HECT	RhoB	unknown	25249323
Smurf2	HECT	H2AX	unknown	31533041
		RNF20	unknown	33097595
TRIP12	HECT	USP7/HAUSP	K48	27800609
UBR5	HECT	ATMIN	unknown	25092319
WWP2	HECT	SOX2	unknown	25042802, 34193614
APC/C^Cdc20^	multimeric RING	MCL-1	unknown	29987118
		BIM	unknown	24871945
APC/C^CDH1^	multimeric RING	CtIP	unknown	25349192
		E2F1	K11	22580462
BRCA1	RING	CtIP	unknown	16818604
BRCA1-BARD1	RING	histone H2A	mono	33589814
		RNA PolⅡ	K6	15886201, 15905410
CHFR	RING	PARP1	K48, K63	23268447
COP1	RING	p53	unknown	16931761
CRL4A^DDB1^	RING	p53	unknown	17967871
		p73	unknown	23085759
CRL4^Cdt2^	RING	p21	unknown	18794347
Cul4B	RING	p53	K48 (no direct evidence)	33524014
		HUWE1	unknown	25883150
CUL4-DDB-ROC1	RING	histone H3, H4	unknown	16678110
FANCL	RING	FANCD2	unknown	17352736
MARCH7	RING	Mdm2	K63	29295817
Mdm2	RING	p53	K48	12507556
		p73	unknown	34716260
Pirh2	RING	p53	K48	12654245
		CHK2	unknown	23449389
		p73	K11, K29, K48, K63	21994467
Rad5	RING	PCNA	K63	18757916
Rad6	RING	PCNA	K63	12226657
Rad18	RING	PCNA	K63	19851286
RFWD3-Mdm2	RING	p53	unknown	20173098
RNF2	RING	H2AX	unknown	21676867
RNF8	RING	MDC1	K63	18006705, 31182912
		histone H1	K63	29127375
		histone H2A, H2AX	K63	22980979
		JMJD2A	K48	22373579
		Ku80	K48	22266820
		PCNA	unknown	18948756
		NBS1	unknown	23115235
		RecQL4	K6, K27, K29	33674555
RNF19A	RING	BARD1	K63	34789768
RNF20/RNF40	RING	histone H2B	unknown	30692271
RNF111	RING	XPC	unknown	23751493
RNF168	RING	histone H2A	K27, K63	25578731, 22980979
		JMJD2A	unknown	22373579
		H2AX	unknown	31533041
SCF^FBXW7^	RING	p53	K48	31346036, 31337255
		p63	unknown	20571051
		XRCC4	K63	26774286
		PLK1	K48	24970797
		SOX9	unknown	27566146
		BLM	K48	26028025
SCF^SKP2^	RING	NBS1	K63	22464731
SCF^β-TrCP^	RING	BIM	unknown	19150432
		CDC25A	unknown	14681206
		CLASPIN	unknown	16885022
SCF^β-TrCP1^	RING	Mdm2	K48	33676897
SCF^β-TrCP2^	RING	Mdm2	K63	33676897
TRIM17	RING	MCL-1	unknown	22976837
TRIM24	RING	p53	unknown	24820418
UHRF1	RING	RIF1	K63	26727879
Peli1	RING-like	NBS1	K63	30952868
PRP19	U-box	RPA	K63	24332808

**Table 3:** Chemical formula of selected compounds targeting the UPS.

Target	Compounds	Chemical Formula
20S Proteasome	Bortezomib	C_19_H_25_BN_4_O_4_
	Carfilzomib	C_40_H_57_N_5_O_7_
	Oprozomib	C_25_H_32_N_4_O_7_S
	Ixazomib	C_14_H_19_BCl_2_N_2_O_4_
E1 enzyme	TAK-243 (MLN7243)	C_19_H_20_F_3_N_5_O_5_S_2_
E2 enzyme	CC0651	C_20_H_21_Cl_2_NO_6_
	NSC697923	C_11_H_9_NO_5_S
	Leucettamol A	C_30_H_52_N_2_O_2_
	Manadosterol A	C_54_H_83_Na_5_O_21_S_5_
	Manadosterol B	C_54_H_84_Na_4_O_18_S_4_
E3 ligase	Nutlin-3a	C_30_H_30_Cl_2_N_4_O_4_
	KRT-232 (AMG 232)	C_28_H_35_Cl_2_NO_5_S
	Milademetan (DS-3032)	C_30_H_34_Cl_2_FN_5_O_4_
	HDM201	C_26_H_24_Cl_2_N_6_O_4_
	ALRN-6924	C_95_H_140_N_20_O_23_
	GDC-0917	C_29_H_36_N_6_O_4_S
	Debio1143 (Xevinapant)	C_32_H_43_N_5_O_4_
	Apcin	C_13_H_14_Cl_3_N_7_O_4_
	TAME	C_14_H_22_N_4_O_4_S
	Oridonin	C_20_H_28_O_6_
	SZL-P1-41	C_24_H_24_N_2_O_3_S
	Longikaurin A	C_20_H_28_O_5_
	Curcumin	C_21_H_20_O_6_
	Dioscin	C_45_H_72_O_16_
	GS143	C_28_H_19_FN_2_O_4_
	Erioflorin	C_19_H_24_O_6_
Deubiquitinase (DUB)	IU1	C_18_H_21_FN_2_O
	IU1-47	C_19_H_23_ClN_2_O
	WP1130	C_19_H_18_BrN_3_O
	HBX 41,108	C_13_H_3_ClN_4_O

## Data Availability

Not applicable.
